# Low lung function and the risk of incident chronic kidney disease in the Malmö Preventive Project cohort

**DOI:** 10.1186/s12882-020-01758-0

**Published:** 2020-04-08

**Authors:** Suneela Zaigham, Anders Christensson, Per Wollmer, Gunnar Engström

**Affiliations:** 1 Department of Clinical Sciences Malmö, Lund University, CRC 60:13, Jan Waldenströms gata 35, S-20502 Malmö, Sweden; 2 Department of Nephrology, Skåne University Hospital, Malmö, Sweden; 3 Department of Translational Medicine, Lund University, Malmö, Sweden

**Keywords:** Lung function, Spirometry, Incidence, Cohort, Chronic kidney disease

## Abstract

**Background:**

Although the prevalence of kidney disease is higher in those with reduced lung function, the longitudinal relationship between low lung function and future risk of chronic kidney disease (CKD) has not been widely explored.

**Methods:**

Baseline lung function was assessed in 20,700 men and 7325 women from 1974 to 1992. Mean age was 43.4 (±6.6) and 47.5 (±7.9) for men and women respectively. Sex-specific quartiles of FEV_1_ and FVC (L) were created (Q4: highest, reference) and the cohort was also divided by the FEV_1_/FVC ratio (≥ or < 0.70). Cox proportional hazards regression was used to determine the risk of incident CKD events (inpatient or outpatient hospital diagnosis of CKD) in relation to baseline lung function after adjustment for various confounding factors.

**Results:**

Over 41 years of follow-up there were 710 and 165 incident CKD events (main diagnosis) in men and women respectively. Low FEV_1_ was strongly associated with future risk of CKD in men (Q1 vs Q4 adjusted HR: 1.46 (CI:1.14–1.89), p-trend 0.002). Similar findings were observed for FVC in men (1.51 (CI:1.16–1.95), p-trend 0.001). The adjusted risks were not found to be significant in women, for either FEV_1_ or FVC. FEV_1_/FVC < 0.70 was not associated with increased incidence of CKD in men or women.

**Conclusion:**

Low FEV_1_ and FVC levels at baseline are a risk factor for the development of future incident CKD in men. Monitoring kidney function in those with reduced vital capacity in early life could help with identifying those at increased risk of future CKD.

## Background

Chronic kidney disease (CKD) has become an important public health issue [1]. It encompasses substantial morbidity and mortality globally along with important economic implications to health systems [1, 2]. It is an independent risk factor for cardiovascular disease (CVD), which in turn is a major cause of the morbidity and mortality associated with CKD [2]. It has been found that even in early stage kidney disease, the prevalence of adverse clinical outcomes is increased [1] and as such, the early identification of risk factors for CKD is key. Some of the important risk factors for CKD include diabetes mellitus (DM), smoking, hypertension and low birth weight [2–4], all of which are factors also known to be associated with low lung function [5–15].

Additionally, low lung function early in life is known to be a powerful risk factor for future CVD and coronary events (CE) and mortality associated with both CVD and CE [16–24]. Establishing the relationship between low lung function early on in life and future CKD could therefore provide an additional strategy to help identify and reduce the risk of at-risk individuals and reduce the overall morbidity and mortality associated with CKD.

Although there has been some cross-sectional research assessing the relationship between low lung function and CKD [25–29], we are aware of few studies assessing the role of lung function in the prediction of incident kidney disease [30, 31], which have found low lung function to be independently associated with CKD progression.

We aimed to use the Malmö Preventive Project (MPP) cohort to assess the longitudinal relationship between low lung function early in life and the future risk of CKD. We have previously used this cohort to show that poor lung function precedes and significantly predicts the development of DM [5]. If the findings of the prospective study by Sumida et al. [30] can be replicated in the present cohort, it will inevitably add further evidence into the rationale for monitoring kidney function in those with low lung function early in life.

## Methods

### Study population

The study population consisted of subjects from the MPP cohort. Screening activities were carried out between 1974 and 1992 in 33,346 subjects (22,444 men and 10,902 women). The attendance rate was over 70%. The aim of the MPP was to screen middle-aged individuals with an aim to offer preventative treatment to any identified high-risk individuals during the screening. Complete birth cohorts for 1921–1949 were invited for a physical examination, laboratory tests, spirometry and a self-administered questionnaire. During 1974–82 men were mostly screened and during 1982–92 women were mostly screened. The screening program was approved by the Health Service Authority of Malmö. Linkage with the national cause of death and patient registers was approved by the Regional Ethics Committee at Lund.

Subjects with prevalent CKD (register recorded diagnosis before the start of the study (*n* = 18), or a baseline estimated glomerular filtration rate (eGFR) < 60 mL/min/1.73m^2^ (*n* = 621)) were excluded at baseline. The CKD classification in this study is based on eGFR alone in accordance with the 2012 KDIGO guidelines, since data on urine albumin is not available from the MPP cohort. Furthermore, it is based on only one eGFR measurement and not two with at least 3 months between (Stage 1 (normal kidney function, GFR ≥90 mL/min/1.73m^2^), stage 2 (mild reduction, GFR 60–89), stage 3a (mild-moderate reduction, GFR 45–59), stage 3b (moderate to severe reduction, GFR 30–44), stage 4 (severe reduction, GFR 15–29), stage 5 (established kidney failure, GFR < 15)). CKD is defined as stage 3 or higher [32].

Spirometry was performed in birth cohorts during most but not all screening time periods (94% of men and 71% of women underwent spirometry), however individuals were not selected based on symptoms or disease. Those with missing information on forced vital capacity (FVC) or forced expiratory volume in 1 s (FEV_1_) were then excluded (*n* = 4410). Subjects with missing information on baseline eGFR (*n* = 87) and those with an outlying value of eGFR (*n* = 1) were excluded. Subjects with missing information on other covariates were also excluded (*n* = 121) or with no follow-up time recorded (n = 1). Those with an erythrocyte sedimentation rate (ESR) ≥50 mm/h were also excluded as this could indicate any specific inflammatory lung pathology (*n* = 62). The final study population consisted of 28,025 subjects (20,700 men and 7325 women).

### Baseline examinations

#### Physical examination

Spirometry was carried out by trained nursing staff using a Spirotron apparatus (Drägerwerk AG, Lübeck, Germany). One acceptable manoeuvre was required. A fixed stadiometer was used to measure height (m) and a balance beam scale to measure weight (kg). Blood pressure (mmHg) was measured after a 10-min rest in the supine position (two measurements taken and mean value recorded). eGFR was calculated using CKD-EPI 2009 (CKD Epidemiology Collaboration) creatinine equation [33].

#### Laboratory assessments

Blood samples were taken after an overnight fast and were analysed at the Department of Clinical Chemistry, Malmö University Hospital using routine methods (serum total cholesterol and whole blood glucose). The Westergren method was used to determine ESR. During the study period, creatinine was measured with the Jaffe method using a Technicon Auto Analyzer II at the Department of Clinical Chemistry at the University Hospital in Malmö. This method was not calibrated to isotope dilution mass spectrometry (IDMS) traceable values. The reference range was 80–115 μmol/L for men and 60–100 μmol/L for women.

#### Questionnaire

A questionnaire was used to establish smoking habits, prevalent diabetes or CVD, antihypertensive medication use and physical activity. Subjects were then classified as never, former or current smokers based on their responses. In men, physical activity was assessed using the following question: *“Are you mostly engaged in sedentary activity in your spare time?”* and in women the following two questions were used: (1) *“Are you engaged in physical activity (e.g. swimming, gymnastics, badminton, tennis, folk dance, running etc.) 1-2 hours per week?”* or (2*) Do you usually get to do light exercise like walking or cycling (or other activities with similar effort) on a regular weekly basis?”.* Low socioeconomic status was defined as Statistics Sweden socioeconomic index (SEI) group 11–36 (unskilled, or skilled manual workers, or low level non-manual workers).

Prevalent CVD was assessed using answers related to the presence of symptoms and signs of CVD on questionnaire or a hospital diagnosis of myocardial infarction or stroke. Prevalent DM was assessed using fasting whole blood glucose ≥6.1 mmol/L (=plasma glucose ≥7.0 mmol/L), self-reported diabetes or diabetes medication according to questionnaire or any prior diagnosis of diabetes in diabetes registers.

### Endpoint ascertainment

CKD was defined using International classification of diseases (ICD) 8, 9 and 10 codes: (ICD-8: codes 582.00 and 582.09, ICD-9: code 585 and ICD-10 codes N18. Additionally any diagnosis of acute kidney disease (AKD) that also subsequently led to a diagnosis of CKD was also defined as CKD (ICD-8 code 583.99, ICD-9 code 586 or ICD-10 code N19 with any of the codes 582.00, 582.09, 585 or N18 in the same individual at a later time point). The endpoints were retrieved from the Swedish inpatient register and the hospital-based outpatient register. The inpatient register has been operating in south of Sweden since 1970 and became nation-wide in 1987. The outpatient register has been operating since 2001. A validation study was performed of the diagnosis of CKD in the inpatient and hospital-based outpatient registers, by two experienced nephrologists. The hospital records were reviewed and validated according to the criteria [34]. The validation showed that the diagnosis of CKD in these registers was correct or had high probability in 94% of the cases [35].

The two endpoints included: (1) Incident CKD - main diagnosis (main diagnosis on either the inpatient register or outpatient register) and (2) Incident CKD (main diagnosis or one of the first three secondary diagnoses). All subjects were followed from baseline examination to the first main CKD event (endpoint 1) or first main or secondary CKD event (endpoint 2), death, emigration or last follow-up date 31st December 2016, whichever came first.

### Statistical analysis

All statistical analyses were carried out using SPSS (IBM Corp. Released 2016. IBM SPSS Statistics for Windows, Version 24.0. Armonk, NY: IBM Corp). Sex- specific quartiles of FEV_1_ and FVC (L) were created (Q4 reference, Q1 = low lung function). Variables with a positively skewed distribution were log-transformed (ESR). To compare baseline characteristics between subjects in quartiles of FEV_1_ or FVC, one-way analysis of variance (ANOVA) and Pearson’s chi-squared test were used. Cox proportional hazards regression was used to assess the incidence of CKD according to quartiles of FEV_1_ and FVC. Adjustments were made for potential confounding factors including age, baseline eGFR, height, body mass index (BMI), smoking status, cholesterol, history of CVD, history of diabetes, systolic blood pressure (BP), physical activity, social class, ESR (log transformed), BP medication and screening year. The proportional hazards assumptions were tested using time-dependent covariate analysis in the adjusted models in SPSS and Kaplan Meier curves.

As the creatinine values were analysed before IDMS was introduced, the serum creatinine values and eGFR used could have resulted in slightly different estimates of eGFR than otherwise would have been if IDMS traceable creatinine values were used. To assess if this would have affected any results, we also peformed a sensitivity analyses by widening the baseline exclusion criteria to eGFR < 70 mL/min/1.73m^2^ to ensure that we did not include any potential cases at baseline by using an older creatinine assay.

## Results

### Baseline characteristics

Baseline characteristics are presented in Tables 1 and 2 for FEV_1_ and in Supplement Tables 1 and 2 for FVC. As lung function decreased (Q1 vs Q4), age, BMI, BP and cholesterol increased, along with the proportion of subjects who were current smokers, physically inactive, on BP medication or had prevalent conditions such as CVD or DM (p-trend < 0.001). Height and eGFR decreased as lung function decreased (p-trend< 0.001). As lung function decreased, the proportion of subjects in low skilled employment increased and the proportion of those in high skilled employment decreased in Q1 vs Q4 (p-trend< 0.001). This trend was seen for quartiles of both FEV_1_ and FVC and in men and women.

**Table 1:** Baseline characteristics in relation to quartiles of FEV_1_: Males (*n* = 20,700)

	Overall	Q4	Q3	Q2	Q1	***P*** value for trend
FEV_1_ (L)	3.52(±0.77)	4.51(±0.42)	3.79(±0.14)	3.31(±0.14)	2.58(±0.44)	–
Number (n)	20,700	4813	5176	5388	5323	–
Age (years)	43.4 (±6.6)	40.0 (±6.0)	42.2 (±6.0)	44.1 (±6.1)	46.8 (±6.4)	< 0.001
Height (m)	1.77(±0.07)	1.81(±0.06)	1.78(±0.06)	1.76(±0.06)	1.74(±0.06)	< 0.001
BMI (kg/m^2^)	24.6 (±3.3)	24.0 (±2.9)	24.5 (±3.1)	24.7 (±3.3)	25.2 (±3.6)	< 0.001
Baseline eGFR (mL/min/1.73m^2^)	88.0(±13.2)	89.4(±13.3)	88.2(±13.0)	87.5(±13.0)	87.1(±13.3)	< 0.001
Current-smokers (%)	49.2	37.9	44.2	51.3	62.3	< 0.001
Physical inactivity (%)	52.5	44.7	50.1	54.1	60.3	< 0.001
Systolic BP (mmHg)	127 (±15)	125 (±13)	126 (±14)	127 (±15)	129 (±16)	< 0.001
Anti-hypertensive medication (%)	3.7	1.9	2.8	3.4	6.5	< 0.001
Cholesterol (mmol/L)	5.59(±1.07)	5.39 (±1.02)	5.56 (±1.03)	5.66 (±1.08)	5.73 (±1.10)	< 0.001
History of CVD	0.9	0.3	0.5	0.9	2.0	< 0.001
History of diabetes	3.2	2.1	2.3	3.5	4.9	< 0.001
Social class (%)						< 0.001
- Low skilled	45.0	38.4	42.2	46.5	52.2	
- High skilled	43.6	52.1	46.9	42.0	34.2	
- Self-employed	8.3	7.8	8.8	8.6	8.0	
- Other	3.1	1.7	2.1	2.9	5.6	

*Q* Quartile, *FEV*_*1*_ Forced expiratory volume in 1 s, *eGFR* Estimated glomerular filtration rate, *BMI* Body mass index, *BP* Blood pressure, *CVD* Cardiovascular disease. Data consist of mean (±standard deviation) unless otherwise stated. Linear by linear association for chi square tests used for *p*-value for categorical variables, ANOVA test for linearity used for *p*-values of continuous variables

**Table 2:** Baseline characteristics in relation to quartiles of FEV_1_: Females (*n* = 7325)

	Overall	Q4	Q3	Q2	Q1	***P*** value for trend
FEV_1_ (L)	2.63(±0.58)	3.31(±0.33)	2.80(±0.08)	2.46(±0.11)	1.88(±0.33)	–
Number (n)	7325	2027	1629	1933	1736	–
Age (years)	47.5 (±7.9)	43.7 (±8.0)	46.1 (±7.9)	48.9 (±7.1)	51.7 (±5.6)	< 0.001
Height (m)	1.64(±0.06)	1.67(±0.05)	1.64(±0.05)	1.62(±0.05)	1.61(±0.06)	< 0.001
BMI (kg/m^2^)	23.9 (±4.0)	23.0 (±3.4)	23.6 (±3.7)	24.2 (±4.1)	24.6 (±4.6)	< 0.001
Baseline eGFR (mL/min/1.73m^2^)	84.1(±13.6)	85.6(±13.9)	84.4(±13.8)	83.7(±13.2)	82.5(±13.3)	< 0.001
Current-smokers (%)	45.6	33.1	38.1	46.8	65.9	< 0.001
Physical inactivity (%) - Missing data (%)	43.3 12.3	35.7 18.6	39.9 15.3	45.2 10.1	53.2 4.4	< 0.001
Systolic BP (mmHg)	123 (±16)	120 (±14)	122 (±15)	124 (±17)	126 (±18)	< 0.001
Anti-hypertensive medication (%)	6.6	3.3	5.5	7.3	10.8	< 0.001
Cholesterol (mmol/L)	5.66(±1.09)	5.36 (±1.01)	5.56(±1.05)	5.77 (±1.08)	5.99 (±1.11)	< 0.001
History of CVD	0.7	0.2	0.2	1.1	1.3	< 0.001
History of diabetes	2.6	1.2	1.4	3.2	4.7	< 0.001
Social class (%)						–
- Low skilled	45.1	37.1	42.4	49.4	52.0	
- High skilled	45.0	55.8	48.4	49.8	34.8	
- Self-employed	2.9	2.8	2.8	2.8	3.1	
- Other	7.1	4.2	6.3	8.1	10.1	

*Q* Quartile, *FEV*_*1*_ Forced expiratory volume in 1 s, *eGFR* Estimated glomerular filtration rate, *BMI* Body mass index, *BP* Blood pressure, *CVD* Cardiovascular disease. Data consist of mean (±standard deviation) unless otherwise stated. Linear by linear association for chi square tests used for *p*-value for categorical variables, ANOVA test for linearity used for *p*-values of continuous variables

### Incidence of CKD

Mean follow-up time was 31 years for men and 30 years for women. There were 710 incident CKD events where CKD was the main diagnosis and 1021 incident CKD events where CKD was either the main or a secondary diagnosis in men. For women these were 165 and 237 events respectively. The hazard ratios (HR) of incident CKD events by quartiles of FEV_1_ for men and women are shown in Tables 3 and 4, respectively. In men, there was an approximately 46% increase in the adjusted risk of incident CKD as the main diagnosis in Q1 of FEV_1_ vs Q4 (reference) (p-trend: 0.002) and a 43% increase in the adjusted risk of incident CKD as the main or secondary diagnosis (p-trend: < 0.001). For quartiles of FVC in men, there was a 51% increase in the adjusted risk of incident CKD as the main diagnosis in Q1 vs Q4 (p-trend: 0.001), and 45% increase in the adjusted risk of incident CKD as the main or secondary diagnosis in Q1 vs Q4 (p-trend: 0.001) (Supplement Table 3). For women, the risk of incident CKD became non-significant in Q1 of FEV_1_ and FVC vs the reference after adjustment for confounding factors (Table 4 and Supplement Table 4).

**Table 3:** Hazard ratios of incident CKD events by quartiles of FEV_1_ in men (*n* = 20,700)

		Q4 (reference)	Q3	Q2	Q1	***P*** value for trend
		4.51(±0.42)	3.79(±0.14)	3.31(±0.14)	2.58(±0.44)	
Number (n)	20,700	4813	5176	5388	5323	–
Incident CKD: Main diagnosis (*n* = 710)	CKD events n (n per 1000 person-years)	130 (0.82)	167 (1.00)	191 (1.16)	222 (1.52)	
	Unadjusted	1.00	1.26 (1.00–1.58)	1.56 (1.25–1.95)	2.40 (1.93–2.98)	< 0.001
	Adjusted^a^	1.00	1.09 (0.86–1.38)	1.19 (0.94–1.52)	1.46 (1.14–1.89)	0.002
Incident CKD: Main or secondary diagnosis (*n* = 1021)	CKD events n (n per 1000 person-years)	183 (1.15)	241 (1.45)	287 (1.74)	310 (2.13)	
	Unadjusted	1.00	1.29 (1.06–1.56)	1.68 (1.40–2.02)	2.41 (2.01–2.89)	0.000
	Adjusted^a^	1.00	1.10 (0.90–1.33)	1.24 (1.02–1.52)	1.43 (1.16–1.77)	< 0.001

^a^Adjustments: Age, baseline eGFR, height, BMI, smoking status, cholesterol, history of CVD, history of diabetes, systolic BP, physical activity, social class, ESR (log transformed), BP medication, screening year

Incident CKD events: includes both inpatient hospitalisations and outpatient diagnosis. Main diagnosis = Primary diagnosis, Main or secondary diagnosis = Primary diagnosis or 1st-3rd secondary diagnosis

**Table 4:** Hazard ratios of incident CKD events by quartiles of FEV_1_ in women (*n* = 7325)

		Q4 (reference)	Q3	Q2	Q1	***P*** value for trend
		3.31(±0.33)	2.80(±0.08)	2.46(±0.11)	1.88(±0.33)	
Number (n)	7325	2027	1629	1933	1736	–
Incident CKD: Main diagnosis (*n* = 165)	CKD events n (n per 1000 person-years)	29 (0.44)	36 (0.70)	51 (0.89)	49 (1.07)	
	Unadjusted	1.00	1.64 (1.01–2.67)	2.26 (1.43–3.57)	3.15 (1.99–4.99)	< 0.001
	Adjusted^a^	1.00	1.21 (0.73–2.01)	1.33 (0.80–2.20)	1.27 (0.73–2.22)	0.403
Incident CKD: Main or secondary diagnosis (*n* = 237)	CKD events n (n per 1000 person-years)	40 (0.61)	52 (1.01)	75 (1.32)	70 (1.54)	
	Unadjusted	1.00	1.73 (1.14–2.61)	2.45 (1.67–3.60)	3.38 (2.29–4.99)	< 0.001
	Adjusted^a^	1.00	1.22 (0.80–1.87)	1.31 (0.86–1.99)	1.22 (0.77–1.94)	0.442

^a^Adjustments: Age, baseline eGFR, height, BMI, smoking status, cholesterol, history of CVD, history of diabetes, systolic BP, physical activity, social class, ESR (log transformed), BP medication, screening year

Incident CKD events: includes both inpatient hospitalisations and outpatient diagnosis. Main diagnosis = Primary diagnosis, Main or secondary diagnosis = Primary diagnosis or 1st-3rd secondary diagnosis

**Fig. 1:**
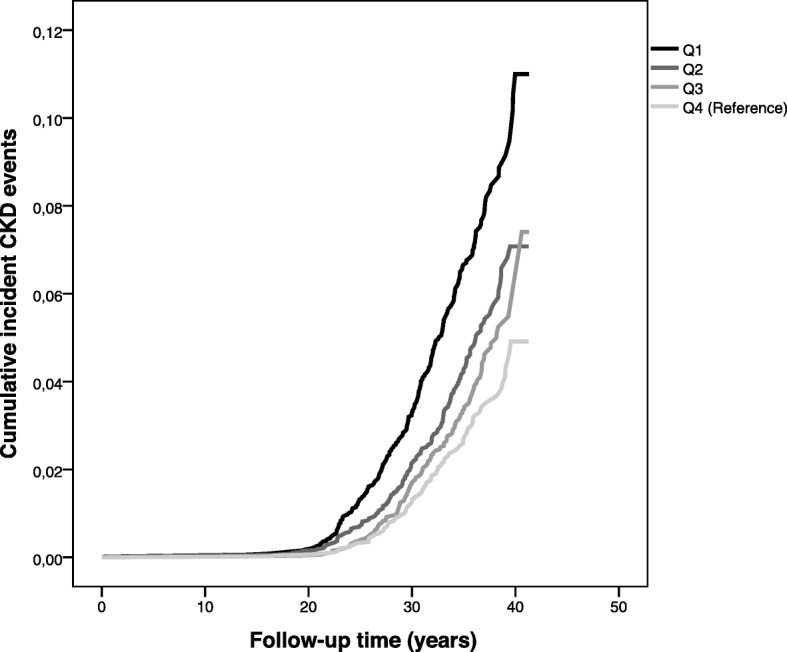
Kaplan Meier curve of incident CKD events by quartiles of FEV_1_ (Q1: lowest lung function, Q4: highest lung function (reference) in men. Incident CKD events were main diagnosis on either inpatient or outpatient registers

**Fig. 2:**
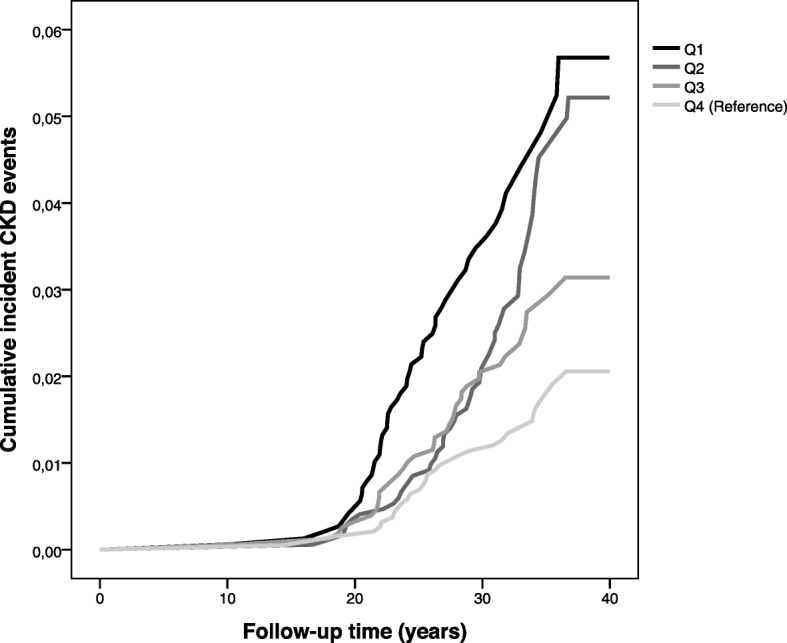
Kaplan Meier curve of incident CKD events by quartiles of FEV_1_ (Q1: lowest lung function, Q4: highest lung function (reference) in women. Incident CKD events were main diagnosis on either inpatient or outpatient registers

Figures 1 and 2 illustrate the differences in cumulative CKD incidence (main CKD diagnosis events only) for quartiles of FEV_1_ in men and women respectively. Differences between the quartiles were tested using the log-rank test, overall pooled and pairwise comparisons. For men, the *p*-value for overall comparisons using the log-rank test was < 0.001. Pairwise comparisons found a *p*-value of < 0.05 for Q1 vs Q2, Q1 vs Q3 and Q1 vs Q4. For women, the p-value for overall comparisons using the log-rank test < 0.001. Pairwise comparisons found a p-value of *p* < 0.05 for Q1 vs Q3 and Q1 vs Q4, but not Q1 vs Q2.

Additionally, there was no increase in the adjusted risk of incident CKD events in those with a ratio of FEV_1_/FVC < 0.70 compared to those with a FEV_1_/FVC ≥ 0.70 at baseline (Supplement Tables 5 and 6)**.**

### Sensitivity analysis

To ensure that we did not include any potential cases at baseline by using an older creatinine assay, we carried out a sensitivity analysis where we widened the baseline exclusion criteria to exclude those with a eGFR < 70 mL/min/1.73^2^. This resulted in an exclusion of a further 1683 subjects from the original cohort of 20,700 men and a further exclusion of 1173 subjects from the original cohort of 7325 women. The results remained significant in men. The adjusted hazard ratio for incident CKD as the main diagnosis for Q1 vs Q4 of FEV_1_ in men was 1.45 (1.10–1.90) (p-trend 0.005) and for incident CKD as main or secondary diagnosis was 1.57 (1.24–1.98) (p-trend < 0.001). The results for women remained non-significant. The adjusted hazard ratio for incident CKD as the main diagnosis for Q1 vs Q4 of FEV_1_ in women was 1.30 (0.67–2.54) (p-trend 0.408) and for incident CKD as main or secondary diagnosis the results again remained non-significant (Q1 vs Q4 (reference): 1.15 (0.67–2.00), p-trend: 0.589).

## Discussion

Although a cross-sectional relationship between low lung function and CKD previously has been found in subjects with chronic obstructive pulmonary disease (COPD) [25, 27, 28], the longitudinal relationship between low lung function early in life and the future risk of incident CKD has not been as widely established Our study investigates this longitudinal relationship in over 28,000 subjects in the MPP cohort. We have found that low FEV_1_ and FVC values at baseline have a strong relationship with future incident CKD events in men but not women, even after adjustment for confounding factors such as diabetes, smoking and BP.

### Comparison with existing literature

The relationship between low lung function and various future health outcomes such as all-cause mortality [23], CVD [20, 24], CE [16, 17, 21] and even DM [5, 8, 9, 11, 36, 37] and COPD [38, 39] has been explored in the past. The mechanisms linking some of these outcomes to low lung function have included chronic inflammation and hypoxia [30]; mechanisms that have also been thought to be linked to the development of CKD [30]. Our findings for incident CKD are consistent with the findings of the recent longitudinal study assessing low lung function at baseline and incident end-stage renal disease (ESRD) and CKD [30]. Although we did not assess ESRD, our findings for incident CKD events were found to be similar; low FVC was found to be a strong predictor of incident CKD. A restrictive lung profile had a stronger association with incident ESRD than an obstructive lung profile at baseline [30]. A population based study by Yoon et al. [26] also found the restrictive lung picture (FVC < 80%) as opposed to an obstructive picture (FEV_1_/VC < 0.70) to be associated with microalbuminuria. However, a recent retrospective cohort study in over 10,000 subjects from Korea [31] found that a 10% decrement in the FEV_1_/FVC ratio in non-smoking subjects was associated with a 35% increase in risk of future CKD after 5 years of follow-up. This is in contrast to our results (Supplement Tables 5 and 6). In our cohort of over 27,000 subjects, the FEV_1_/FVC ratio was not associated with incidence of CKD. This difference may exist due to the way CKD was defined as an outcome; in the retrospective study [31], a visit during follow-up for a health assessment was used to obtain eGFR measurements, which was then used to define the presence of CKD. In our prospective study design, subjects were followed up for extensive time periods and outcomes were ascertained as a first hospitalisation or outpatient visit due to CKD (or AKD which then led to CKD) with a diagnosis stated using ICD coding for CKD. Therefore, outcomes in our cohort may represent the more severe end of the disease spectrum, as subjects would need to have presented to secondary healthcare and been diagnosed by a health professional.

We have previously found that low FEV_1_ and FVC are strong predictors of DM in the MPP cohort [5]. In the study, again there was no link between an obstructive lung picture at baseline and future risk of DM. As DM is a risk factor for the development of CKD, DM could potentially mediate the relationship between lung function and CKD. However, the prevalence of DM was low in the MPP cohort and the risk of CKD was increased in men with low FVC or low FEV_1_, even after adjustment for prevalent DM, which was defined by baseline fasting plasma glucose ≥7.0 mmol/L, self-reported diabetes, questionnaire reporting DM medication or any prior diagnosis of DM recorded on relevant registers.

Lung function and CKD share common risk factors [31] such as, smoking, low birth weight, age, hypertension and obesity. The risk of CKD in relation to baseline FEV_1_ or FVC remained significant in men even after adjusting for smoking, age, systolic BP and BMI. The role of early life factors such as low birth weight however could not be explored, and could potentially explain some of the association between having reduced lung function and development of CKD in adult life.

Previously, an association has also been found between lung function decline on spirometry and future incident arterial hypertension [15, 40, 41], which is a well-known important pathway in the pathogenesis of CKD. Another proposed mechanism that may link lung function to CKD is systemic inflammation. Those that have low levels of lung function may exhibit underlying lung pathology associated with low-grade chronic inflammation. Persistent low grade inflammation is thought to be a “hall-mark feature” of CKD, where the role of inflammation in CKD progression and subsequent morbidity and mortality is well established [42]. Although we adjusted for ESR in our analysis and excluded those with a value of ≥50 mm/h from the analysis, we did not adjust for various other inflammatory markers that could potentially play a role in the association between lung function and CKD. Therefore, we cannot fully exclude the potential effect of low-grade inflammation linking low levels of lung function to increased CKD risk.

We found a significant relationship between low FEV_1_ and incidence of CKD in men, but this relationship was non-significant after risk factor adjustment in women. The reason for this difference is unclear. As expected, incidence of CKD was higher in men than in women and since more men than women were included, the non-significant finding in women could be a result of lower statistical power. However, incidence of risk factors over a life span, such as diabetes and hypertension are also usually higher in men than in women. This could also possibly result in higher risks for men with poor lung function.

### Study limitations

There are some methodological considerations for this study. The study was carried out before the current guidelines for spirometry were available. Therefore, no nose clips were used during spirometry measurements and only one acceptable manoeuvre was required, which could potentially have resulted in measurement errors. The long follow-up time for this study has its drawbacks along with its advantages. Many baseline characteristics may have changed over 41 years of follow-up. Interventions such as medication use for those with poorest lung function may have included corticosteroids which can potentially increase the risk of DM, in turn increasing the potential risk of diabetic nephropathy. However, it has been found that while this may be possible with oral corticosteroid use, inhaled corticosteroids such as budesonide are not associated with DM or hyperglycaemia in COPD or asthma patients treated with it [43]. Smoking rates have been known to have declined in Sweden [44], therefore it is very likely that many of the “baseline smokers” quit smoking during follow-up. However, this would if anything, bias our results towards the null. BMI also could have changed over this extensive follow-up period, which in turn could affect the risks of DM and hypertension, which are known risk factors for CKD. Individuals with hypertension, hyperglycemia, or hyperlipidemia were referred to an outpatient clinic for further risk factor evaluation and possible treatment. The beneficial effect of any intervention is however questionable. A study comparing the long term outcomes of the MPP (mortality and cardiovascular morbidity) found that risk factor screening for those who were invited to take part in the project did not result in a reduction in the total mortality [45].

The follow-up of CKD was based on national hospital based registers. One limitation is that cases that would have presented to primary care were missed. The outpatient register began in 2001, so additionally any CKD cases that would have also presented to outpatient care prior to this date would also have been missed.

In long-term follow-up studies, it is inevitable that creatinine is measured with methods not traceable to IDMS calibration, which was the case in our study. However, it was the same method throughout the study with stable reference values. No available data on urinary albumin-creatinine ratio make it impossible for characterisation into CKD stages 1 and 2 at baseline. Therefore, CKD was defined as eGFR < 60 mL/min/1.73m^2^ for exclusion at baseline as we had no information about albuminuria. However, even after widening the baseline exclusion criteria to eGFR < 70 mL/min/1.73m^2^ (in order to overcome the potential issue of incomplete exclusion of prevalent CKD cases due to creatinine measurements not traceable to IDMS), we found the results remained largely unchanged. Therefore, we find it unlikely that the creatinine values used for the calculation of eGFR and baseline exclusions in our original analysis would have affected the results.

## Conclusion

Low FEV_1_ and FVC values in early life are strongly associated with future incident CKD events in men even after adjustment for confounding factors such as diabetes, smoking and BP. Monitoring kidney function in those with reduced vital capacity in early life could help with identifying those at increased risk of future CKD. Lung function could therefore have a potential role in CKD risk prediction.

## Supplementary information

**Additional file 1: Table S1.** Baseline characteristics in relation to quartiles of FVC: Males (*n* = 20,700). **Table S2.** Baseline characteristics in relation to quartiles of FVC: Females (*n* = 7325). **Table S3.** Hazard ratios of incident CKD events by quartiles of FVC in men (*n* = 20,700). **Table S4.** Hazard ratios of incident CKD events by quartiles of FVC in women (*n* = 7325). **Table S5.** Hazard ratios of incident CKD events by FEV_1_/VC ratio in men (*n* = 20,700). **Table S6.** Hazard ratios of incident CKD events by FEV_1_/VC ratio in women (*n* = 7325).

## Data Availability

The MPP steering committee coordinates research using the MPP database. The data base is open for applications for research projects. Contact: Anders Dahlin, PhD, data manager, Email: Anders.Dahlin@med.lu.se

## Abbreviations

CKD: Chronic kidney disease
CVD: Cardiovascular disease
DM: Diabetes mellitus
CE: Coronary events
MPP: Malmö Preventive Project
eGFR: Estimated glomerular filtration rate
FVC: Forced vital capacity
FEV_1_: Forced expiratory volume in 1 s
ESR: Erythrocyte sedimentation rate
IDMS: Isotope dilution mass spectrometry
SEI: Statistics Sweden socioeconomic index
ICD: International classification of diseases
AKD: Acute kidney disease
ANOVA: One-way analysis of variance
BMI: Body mass index
Q: Quartile
BP: Blood pressure
HR: Hazard ratio
COPD: Chronic obstructive pulmonary disease
ESRD: End-stage renal disease
