# Supplementary material for: Low lung function and the risk of incident chronic kidney disease in the Malmö Preventive Project cohort
Source: BMC Nephrol. 2020 Apr 8;21:124. doi: 10.1186/s12882-020-01758-0 (PMC7144045; doi:10.1186/s12882-020-01758-0)
Supplement: Supplementary file 1 — Additional file 1: Table S1. Baseline characteristics in relation to quartiles of FVC: Males (n = 20,700). Table S2. Baseline characteristics in relation to quartiles of FVC: Females (n = 7325). Table S3. Hazard ratios of incident CKD events by quartiles of FVC in men (n = 20,700). Table S4. Hazard ratios of incident CKD events by quartiles of FVC in women (n = 7325). Table S5. Hazard ratios of incident CKD events by FEV1/VC ratio in men (n = 20,700). Table S6. Hazard ratios of incident CKD events by FEV1/VC ratio in women (n = 7325). [file 12882_2020_1758_MOESM1_ESM.docx]

**Supplement Table 1: Baseline characteristics in relation to quartiles of FVC: Males (n= 20,700)**

|  | **Overall** | **Q4** | **Q3** | **Q2** | **Q1** | **P value for trend** |
| --- | --- | --- | --- | --- | --- | --- |
| FVC (Litres) | 4.54 (±0.91) | 5.71 (±0.51) | 4.84 (±0.17) | 4.26 (±0.17) | 3.39 (±0.49) | - |
| Number (n) | 20 700 | 4861 | 5362 | 5433 | 5044 | - |
| Age (years) | 43.4 (±6.6) | 40.7 (±6.1) | 42.3 (±6.1) | 44.0 (±6.3) | 46.4 (±6.5) | <0.001 |
| Height (m) | 1.77 (±0.07) | 1.82 (±0.06) | 1.78 (±0.06) | 1.76 (±0.06) | 1.73 (±0.06) | <0.001 |
| BMI (kg/m^2^) | 24.6 (±3.3) | 24.1 (±2.9) | 24.4 (±3.1) | 24.8 (±3.3) | 25.2 (±3.7) | <0.001 |
| Baseline eGFR (mL/min/1.73m^2^) | 88.0 (±13.2) | 88.9 (±13.1) | 88.2 (±13.2) | 87.8 (±13.0) | 87.3 (±13.4) | <0.001 |
| Current-smokers (%) | 49.2 | 41.0 | 45.1 | 51.6 | 59.0 | <0.001 |
| Physical inactivity (%) | 52.5 | 44.9 | 50.8 | 54.2 | 59.9 | <0.001 |
| Systolic BP (mmHg) | 127 (±15) | 125 (±13) | 126 (±14) | 127 (±15) | 129 (±17) | <0.001 |
| Anti-hypertensive medication (%) | 3.7 | 2.0 | 2.6 | 4.2 | 6.0 | <0.001 |
| Cholesterol (mmol/L) | 5.59 (±1.07) | 5.42 (±1.02) | 5.55 (±1.03) | 5.66 (±1.08) | 5.71 (±1.10) | <0.001 |
| History of CVD | 0.9 | 0.3 | 0.6 | 0.9 | 1.9 | <0.001 |
| History of diabetes | 3.2 | 1.9 | 2.5 | 3.3 | 5.3 | <0.001 |
| Social class (%)  - Low skilled  - High skilled  - Self-employed  - Other | 45.0  43.6  8.3  3.1 | 37.3  53.2  7.9  1.5 | 42.6  46.9  8.3  2.2 | 47.7  40.9  8.6  2.8 | 52.2  33.6  8.3  5.9 | <0.001 |

Q: Quartile, FVC: Forced vital capacity, eGFR: estimated glomerular filtration rate, BMI: Body mass index, BP: Blood pressure, CVD: cardiovascular disease. Data consist of mean (±standard deviation) unless otherwise stated. Linear by linear association for chi square tests used for p-value for categorical variables, ANOVA test for linearity used for p-values of continuous variables

**Supplement Table 2: Baseline characteristics in relation to quartiles of FVC: Females (n=7325)**

|  | **Overall** | **Q4** | **Q3** | **Q2** | **Q1** | **P value for trend** |
| --- | --- | --- | --- | --- | --- | --- |
| FVC (litres) | 3.28 (±0.67) | 4.08 (±0.40) | 3.44 (±0.11) | 3.06 (±0.11) | 2.42 (±0.38) | - |
| Number (n) | 7325 | 1977 | 1834 | 1796 | 1718 | - |
| Age (years) | 47.5 (±7.9) | 44.5 (±7.9) | 46.5 (±7.9) | 48.4 (±7.6) | 51.2 (±6.0) | <0.001 |
| Height (m) | 1.64 (±0.06) | 1.68 (±0.05) | 1.64 (±0.05) | 1.62 (±0.05) | 1.60 (±0.06) | <0.001 |
| BMI (kg/m^2^) | 23.9 (±4.0) | 23.1 (±3.4) | 23.6 (±3.7) | 24.0 (±4.1) | 24.8 (±4.7) | <0.001 |
| Baseline eGFR (mL/min/1.73m^2^) | 84.1 (±13.6) | 85.0  (±13.9) | 83.9  (±13.4) | 84.2  (±13.6) | 83.1  (±13.4) | <0.001 |
| Current-smokers (%) | 45.6 | 36.0 | 39.6 | 48.8 | 59.7 | <0.001 |
| Physical inactivity (%)   - Missing data (%) | 43.3  12.3 | 34.2  19.9 | 41.4  13.1 | 46.2  9.0 | 52.7  5.1 | <0.001 |
| Systolic BP (mmHg) | 123 (±16) | 120 (±14) | 122 (±16) | 124 (±17) | 127 (±18) | <0.001 |
| Anti-hypertensive medication (%) | 6.6 | 3.7 | 5.3 | 7.2 | 10.8 | <0.001 |
| Cholesterol (mmol/L) | 5.66 (±1.09) | 5.40 (±1.00) | 5.58 (±1.07) | 5.73 (±1.07) | 5.97 (±1.12) | <0.001 |
| History of CVD | 0.7 | 0.3 | 0.3 | 1.1 | 1.3 | <0.001 |
| History of diabetes | 2.6 | 1.4 | 1.5 | 2.4 | 5.3 | <0.001 |
| Social class (%)  - Low skilled  - High skilled  - Self-employed  - Other | 45.1  45.0  2.9  7.1 | 37.1  55.8  2.7  4.4 | 43.7  47.4  2.9  6.0 | 49.1  40.3  3.1  7.5 | 51.5  34.7  2.9  11.0 | <0.001 |

Q: Quartile, FVC: Forced vital capacity, eGFR: estimated glomerular filtration rate, BMI: Body mass index, BP: Blood pressure, CVD: cardiovascular disease. Data consist of mean (±standard deviation) unless otherwise stated. Linear by linear association for chi square tests used for p-value for categorical variables, ANOVA test for linearity used for p-values of continuous variables

**Supplement Table 3: Hazard ratios of incident CKD events by quartiles of FVC in men (n=20,700)**

|  |  | **Q4**  **(reference)** | **Q3** | **Q2** | **Q1** | **P value for trend** |
| --- | --- | --- | --- | --- | --- | --- |
|  |  | 5.71 (±0.51) | 4.84 (±0.17) | 4.26 (±0.17) | 3.39 (±0.49) |  |
| Number (n) | 20 700 | 4861 | 5362 | 5433 | 5044 | - |
|  |  |  |  |  |  |  |
| Incident CKD:  Main diagnosis  (n=710) | CKD events n (n per 1000 person-years) | 131 (0.82) | 172 (1.00) | 196 (1.18) | 211 (1.51) |  |
|  | Unadjusted | 1.00 | 1.25 (0.99-1.56) | 1.56 (1.25-1.95) | 2.32 (1.86-2.88) | <0.001 |
|  | Adjusted* | 1.00 | 1.13 (0.89-1.42) | 1.25 (0.98-1.59) | 1.51 (1.16-1.95) | 0.001 |
|  |  |  |  |  |  |  |
|  |  |  |  |  |  |  |
|  |  |  |  |  |  |  |
| Incident CKD:  Main or secondary diagnosis  (n=1021) | CKD events n (n per 1000 person-years) | 189 (1.19) | 255 (1.49) | 284 (1.71) | 293 (2.11) |  |
|  | Unadjusted | 1.00 | 1.28 (1.06-1.55) | 1.58 (1.31-1.89) | 2.25 (1.88-2.71) | <0.001 |
|  | Adjusted* | 1.00 | 1.15 (0.95-1.39) | 1.24 (1.02-1.51) | 1.45 (1.17-1.80) | 0.001 |
|  |  |  |  |  |  |  |
|  |  |  |  |  |  |  |
|  |  |  |  |  |  |  |

*Adjustments: Age, baseline eGFR, height, BMI, smoking status, cholesterol, history of CVD, history of diabetes, systolic BP, physical activity, social class, ESR (log transformed), BP medication, screening year

Incident CKD events: includes both inpatient hospitalisations and outpatient diagnosis. Main diagnosis = Primary diagnosis, Main or secondary diagnosis= Primary diagnosis or 1^st^-3^rd^ secondary diagnosis.

**Supplement Table 4: Hazard ratios of incident CKD events by quartiles of FVC in women (n= 7,325)**

|  |  | **Q4**  **(reference)** | **Q3** | **Q2** | **Q1** | **P value for trend** |
| --- | --- | --- | --- | --- | --- | --- |
|  |  | 4.08 (±0.40) | 3.44 (±0.11) | 3.06 (±0.11) | 2.42 (±0.38) |  |
| Number (n) | 7325 | 1977 | 1834 | 1796 | 1718 | - |
|  |  |  |  |  |  |  |
| Incident CKD:  Main diagnosis  (n=165) | CKD events n (n per 1000 person-years) | 33 (0.52) | 37 (0.65) | 45 (0.85) | 50 (1.08) |  |
|  | Unadjusted | 1.00 | 1.29 (0.81-2.06) | 1.79 (1.14-2.80) | 2.59 (1.67-4.03) | <0.001 |
|  | Adjusted* | 1.00 | 1.02 (0.63-1.66) | 1.14 (0.69-1.86) | 1.10 (0.65-1.87) | 0.657 |
|  |  |  |  |  |  |  |
|  |  |  |  |  |  |  |
|  |  |  |  |  |  |  |
| Incident CKD:  Main or secondary diagnosis  (n=237) | CKD events n (n per 1000 person-years) | 44 (0.69) | 55 (0.96) | 65 (1.22) | 73 (1.59) |  |
|  | Unadjusted | 1.00 | 1.45 (0.97-2.15) | 1.97 (1.34-2.89) | 2.93 (2.01-4.26) | <0.001 |
|  | Adjusted* | 1.00 | 1.12 (0.73-1.67) | 1.17 (0.77-1.78) | 1.17 (0.75-1.83) | 0.492 |
|  |  |  |  |  |  |  |
|  |  |  |  |  |  |  |
|  |  |  |  |  |  |  |

*Adjustments: Age, baseline eGFR, height, BMI, smoking status, cholesterol, history of CVD, history of diabetes, systolic BP, physical activity, social class, ESR (log transformed), BP medication, screening year.

Incident CKD events: includes both inpatient hospitalisations and outpatient diagnosis. Main diagnosis = Primary diagnosis, Main or secondary diagnosis= Primary diagnosis or 1^st^-3^rd^ secondary diagnosis.

**Supplement table 5: Hazard ratios of incident CKD events by FEV_1_/VC ratio in men (n=20,700)**

|  |  | **FEV_1_/FVC ≥0.70** | **FEV_1_/VC <0.70** | **P value** |
| --- | --- | --- | --- | --- |
|  |  |  |  |  |
| Incident CKD:  Main diagnosis |  |  |  |  |
| (n=710) | Unadjusted | 1.00 | 1.20 (0.99-1.47) | 0.067 |
|  | Adjusted* | 1.00 | 1.03 (0.84-1.26) | 0.793 |
|  |  |  |  |  |
|  |  |  |  |  |
|  |  |  |  |  |
| Incident CKD:  Main or secondary diagnosis |  |  |  |  |
| (n=1021) | Unadjusted | 1.00 | 1.34 (1.14-1.57) | 0.000 |
|  | Adjusted* | 1.00 | 1.12 (0.95-1.32) | 0.165 |
|  |  |  |  |  |
|  |  |  |  |  |
|  |  |  |  |  |

*Adjustments: Age, baseline eGFR, height, BMI, smoking status, cholesterol, history of CVD, history of diabetes, systolic BP, physical activity, social class, ESR (log transformed), BP medication, screening year

Incident CKD events: includes both inpatient hospitalisations and outpatient diagnosis. Main diagnosis = Primary diagnosis, Main or secondary diagnosis= Primary diagnosis or 1^st^-3^rd^ secondary diagnosis.

**Supplement table 6: Hazard ratios of incident CKD events by FEV_1_/VC ratio in women (n=7,325)**

|  |  | **FEV_1_/FVC ≥0.70** | **FEV_1_/VC <0.70** | **P value** |
| --- | --- | --- | --- | --- |
|  |  |  |  |  |
| Incident CKD:  Main diagnosis |  |  |  |  |
| (n=165) | Unadjusted | 1.00 | 1.76 (1.14-2.74) | 0.012 |
|  | Adjusted* | 1.00 | 1.25 (0.80-1.98) | 0.330 |
|  |  |  |  |  |
|  |  |  |  |  |
|  |  |  |  |  |
| Incident CKD:  Main or secondary diagnosis |  |  |  |  |
| (n=237) | Unadjusted | 1.00 | 1.73 (1.19-2.51) | 0.004 |
|  | Adjusted* | 1.00 | 1.19 (0.81-1.75) | 0.369 |
|  |  |  |  |  |
|  |  |  |  |  |
|  |  |  |  |  |

*Adjustments: Age, baseline eGFR, height, BMI, smoking status, cholesterol, history of CVD, history of diabetes, systolic BP, physical activity, social class, ESR (log transformed), BP medication, screening year

Incident CKD events: includes both inpatient hospitalisations and outpatient diagnosis. Main diagnosis = Primary diagnosis, Main or secondary diagnosis= Primary diagnosis or 1^st^-3^rd^ secondary diagnosis.
